# The Arf GEF GBF1 integrates inputs from its product, Arf4·GTP, and the Golgi influx of sensory membrane cargo to activate Arf4 in ciliary trafficking

**DOI:** 10.1186/2046-2530-4-S1-P24

**Published:** 2015-07-13

**Authors:** D Deretic, J Wang

**Affiliations:** 1 Surgery/Ophthalmology, University of New Mexico, Albuquerque, NM, USA

## Objective

Primary cilia in biology and disease are of considerable importance, however, molecular mechanisms for specific targeting of sensory receptors to primary cilia are still unknown. In the present study we wanted to determine if the small GTPase Arf4 is activated by the Arf guanine nucleotide exchange factor (GEF) GBF1 in ciliary trafficking, and examine the role of the ciliary cargo, rhodopsin, in the activation of Arf4.

## Methods

The distribution of GBF1 and its interactions with rhodopsin and Arf4 were examined by pulse-chase experiments, retinal subcellular fractionation, confocal microscopy and the Proximity Ligation Assay (PLA), which detects protein-protein interactions in situ.

## Results

The Arf GEF GBF1 co-localized with the Rab6 GTPase at the photoreceptor *trans*-Golgi. The PLA analysis revealed that Arf4 and rhodopsin interact with GBF1, nearly exclusively at the Golgi/TGN. The GBF1 GEF activity was essential for the GBF1-rhodopsin-Arf4 interaction, since its selective inhibitor Golgicide A (GCA) caused a significant decrease in all but the GBF1-ASAP1 interactions. GCA specifically inhibited rhodopsin delivery to the cilia, but, in contrast to BFA, it had no effect on the Golgi morphology. GST-pulldowns of purified rhodopsin with DCB-HUS and Sec7-HDS1 domains of GBF1, with or without recombinant Arf4 pre-loaded with GTPgS or GDPbS, showed that rhodopsin and activated Arf4 interact with DCB-HUS domain, whereas GDP-bound Arf4 interacts with Sec7-HDS1 domain.

## Conclusions

Our findings suggest that GBF1 acts both as a GEF and an effector of Arf4. GBF1 integrates the input from both the influx of rhodopsin into the Golgi/TGN and the activated Arf4, to create a pool of activated Arf4 surrounding the nascent buds that generate ciliary-targeted rhodopsin transport carriers. Thus, the progression of newly synthesized protein cargo through the Golgi coordinates the recruitment and activation of Arf and Rab GTPases allowing their ordered succession en route to cilia.

